# Endocytosis of Fgf8 Is a Double-Stage Process and Regulates Spreading and Signaling

**DOI:** 10.1371/journal.pone.0086373

**Published:** 2014-01-20

**Authors:** Charanya Rengarajan, Alexandra Matzke, Luisa Reiner, Véronique Orian-Rousseau, Steffen Scholpp

**Affiliations:** Karlsruhe Institute of Technology (KIT), Institute of Toxicology and Genetic (ITG), Karlsruhe, Germany; Karlsruhe Institute of Technology, Germany

## Abstract

Tightly controlled concentration gradients of morphogens provide positional information and thus regulate tissue differentiation and morphogenesis in multicellular organisms. However, how such morphogenetic fields are formed and maintained remains debated. Here we show that fibroblast growth factor 8 (Fgf8) morphogen gradients in zebrafish embryos are established and maintained by two essential mechanisms. Firstly, Fgf8 is taken up into the cell by clathrin-mediated endocytosis. The speed of the uptake rate defines the range of the morphogenetic gradient of Fgf8. Secondly, our data demonstrate that after endocytosis the routing of Fgf8 from the early endosome to the late endosome shuts down signaling. Therefore, intracellular endocytic transport regulates the intensity and duration of Fgf8 signaling. We show that internalization of Fgf8 into the early endosome and subsequent transport towards the late endosome are two independent processes. Therefore, we hypothesize that Fgf8 receiving cells control both, the propagation width and the signal strength of the morphogen.

## Introduction

The family of fibroblast growth factors (Fgfs) is currently believed to consist of 23 structurally related polypeptides controlling a wide range of biological functions [1]. The most important roles for Fgf action are during development and regeneration when they regulate cell growth, migration, and differentiation [2], [3]. They are expressed in a strict temporal and spatial pattern during embryonic differentiation and wound healing processes. As paracrine signaling molecules, Fgfs are released from a localized source and form signaling gradients in the neighboring tissues. Fgfs induce concentration-dependent responses in the target cells and therefore they have been postulated to act as morphogens (reviewed in [4]). Indeed, in the receiving cells the biological action of Fgfs is exerted through binding to and activation of high-affinity cell surface Fgf receptors (Fgfrs) that have intrinsic tyrosine kinase activity [5]. Besides PLC-g/protein kinase (PK)C and PI3K/Akt, Ras/MAPK is the major downstream signaling pathway activated by Fgfs, which leads to the phosphorylation of Erk [6]. After activation, internalization and degradation of the Fgf-Fgfr complex is an important regulatory mechanism to restore basal levels of signaling.

Fgf8 is one member of the Fgf family with key inductive functions during development of neural ectoderm, mesoderm and limb formation [7], [8], [9], [10], [11]. In adult, deregulated Fgf8 signaling is involved in a variety of disorders such as the Kallman syndrome as well as certain malignancies [12]. Fgf8 exerts its function mainly via activation of Fgfr1 and Fgfr3 [13], [14], [15]. Endocytosis and degradation of the active Fgf8-Fgfr complex is an important mechanism in down-regulation of signaling and maintenance of the morphogenetic field [16], [17].

One internalization route for growth factors such as Fgfs is via Clathrin mediated endocytosis (CME) a major mechanism for controlled cargo uptake which can be subdivided into several steps [18]: Morphologically, the first stage of vesicle budding involves the formation of a membrane invagination called a pit, to which cargo such as activated receptors are recruited to the membrane [19]. The following vesicle scission depends on the formation of a ring by the mechanochemical enzyme dynamin [20]. Once detached from the parent membrane, the clathrin coat is disassembled from its lattice arrangement back to clathrin triskelia by the ATPase heat shock cognate 70 (Hsc70; [21], [22], which also brings clathrin back to the cell membrane for the next cycle of vesicle formation. Hsc70 mediated dismantling of the clathrin coat allows the vesicle to travel to and fuse with the early endosome and therefore defines an important step of the intracellular endocytic route. Therefore, blockage of Hsc70 function has been suggested as a specific way to interfere with CME. Indeed, loss of Hsc70 function leads to inhibition of transferrin uptake and sorting suggesting that Hsc70 mediated dismantling of the clathrin coat is linked subsequently to endocytosis [23]. In addition, in Hsc70-4^R447H^ mutant fruit flies, it has been shown, that depletion in uncoating activity correlates with decreased endocytosis [24].

The identity of the uptake mechanism for Fgf8 has been a matter of some controversy [17], [25], [26]. After endocytosis, activated Fgfr complexes enter Rab5 positive early endosomes [16], [27], [28] as expected for internalized receptors [29]. Subsequent to their presence in early endosomes, Fgf8 is sorted to Rab7 positive late endosomes and lysosomes for degradation in zebrafish [16], [17]. Therefore the process of degradation of the morphogen by the receiving cells and subsequent limitation of the signaling range has been termed the ‘restrictive clearance model’ [16].

Although some aspects of Fgf8 internalization route are understood, the factors that execute key functions during specific steps of Fgf8 endocytosis are still unclear. Furthermore, it is not fully understood how endocytosis regulates Fgf8 signaling and it is also unclear whether the signals emanate from the membrane and/or from internalized ligand-receptor complexes. Therefore, localization of the internalization route and subsequent identification of the signaling compartments is crucial.

Here the molecular machinery involved in CME of Fgf8 was characterized and the influences of endocytosis on Fgf8 signaling were examined. Therefore, different steps in internalization – the clathrin mediated uptake and the transport from early to late endosomes- were analyzed. Firstly, we analyzed whether Hsc70 controls the endocytosis of Fgf8 by regulating CME. We show that knock-down of Hsc70 leads to a reduction in Fgf8 internalization and subsequently to an increase in long-range signaling of Fgf8 in zebrafish. Consistently, overexpression of Hsc70 leads to enhanced internalization of Fgf8 into early endosomes. Furthermore, we find that Fgf8 displays a decreased signaling activity in the early endosome compared to the plasma membrane. Finally we show that Fgf8 signaling is terminated by transport of the ligand to the late endosome. Therefore, we conclude that the receiving cells control Fgf8 signaling at two independent levels: firstly the dynamic of CME is important for regulating the activity range of Fgf8, and secondly the rate of transport from early endosomes to late endosomes determines Fgf8 signaling strength.

## Materials and Methods

All zebrafish husbandry and experimental procedures were performed in accordance with the German law on Animal Protection and were approved by Local Animal-Protection Committee (Regierungspräsidium Karlsruhe, Az.35-9185.64) and the Karlsruhe Institute of Technology (KIT).

### Fish Maintenance

Breeding zebrafish (*Danio rerio*) were maintained at 28°C on a 14-h light/10-h dark cycle [30]. The data we present in this study were acquired from analysis of wild-type zebrafish of ITG (AB_2_O_2_) and the transgenic zebrafish line Tg(Dusp6:EGFP)^pt6^, which was received from Michael Tsang.

### Injections

pCS2+Fgf8 GFP plasmid [31] and pcDNA3.1DynK44A (Sandy Schmid), pCS2+Rab5 and the pCS2+Rab5-mCherry plasmid were linearized using NOT1 and transcribed with Sp6 Message Machine Kit (Ambion). *Hsc70* full length coding sequence was amplified with primer pairs (forward/reverse); 5′-TGG TGG CAC TTT TGA TGT GT-3′/5′-TCC CTC TCT GCA GTC TGG TT-3′ from a zebrafish cDNA library at stage 24 h. The Hsc70 full-length cDNA was cloned in the expression vector pCS2+. The plasmid was then also linearized with NOT1 and transcribed using SP6 Message Machine Kit (Ambion).

### Morpholino Oligomers

For transient knock-down of gene expression, Morpholino-antisense oligomers (MO, Gene Tools), *hsc70* MO1 (5′ATAAAACAGAGATGGATGAAGATGC 3′) and *hsc70* MO2 (5′ AGCTGGTCCCTTGGACATTGTGTCA 3′) were used at a concentration of 0.5 mM. A volume of 1–2 nl was injected per embryo. The injection of MO oligomers was performed in the yolk cell close to the blastomeres at one to two-cell stage.

### Immunoblot Experiments

For transient knock-down, Hsc70 MO at a concentration 0.5 mM, was injected at the one- to two-cell stages. Samples of 30 embryos were collected at 28hpf, homogenized and dissolved in 1% triton lysis buffer. Protein solution was then eluted with Lammli buffer and heated to 95°C for 5 min and subjected to SDS-PAGE.

For immunoblot, a Hsc70 polyclonal antibody (Abcam, ab79857) was used at a dilution 1∶50,000. This was followed by incubation with anti-mouse IgG-HRP (Dako, PO447) at a dilution 1∶10,000. Chemiluminescence detection was performed using ECL Western Blotting Substrate Kit (Pierce).

### Immunoblot Experiments with Cell Lysates

HEK 293 cells were cultivated to 70–80% confluency before transfection. For transfection of Fgf8 and Hsc70 cells were seeded at 2×10^6^ cells in a 10cm plate. After 24 hours cells were transfected according to the manufacturer’s protocol using Promofectin (Promocell). Briefly two mixtures containing DNA/serum free DMEM and Promofectin/serum-free DMEM were combined incubated for 20 min at RT and added to the cells. 24 hrs after transfection the culture medium was replaced and Bafilomycin A1 was added. 24 hrs later the cells were lysed in sodium dodecyl sulfate (SDS)–sample buffer containing 100 mM dithiothreitol (DTT) and subjected to Western blot analysis. Activated Erk was monitored using an antibody against phosphorylated Erk (phospho-p44/42, Cell Signaling). For the loading control the membrane was stripped (62.5 mM Tris, pH 6.8, 2% SDS, 0.8% DTT) and reprobed with an Erk antibody (Erk 1 (K-23) Santa Cruz). Blots were stained using the enhanced chemiluminescence system (Thermo Fisher Scientific). The quantification of proteins bands in Western blot analysis was performed using the program ImageJ.

### Quantification and Statistical Analysis

The statistical analysis was performed on three independent experiments. All quantifications are given as mean plus standard deviation.

### Whole-mount *in situ* Hybridization

Whole-mount mRNA *in situ* hybridization was carried out as described previously [32]. For visualizing cell nuclei, embryos were fixed in 4% paraformaldehyde/PBS at room temperature for 2 hours. Digoxygenin- and fluorescein-labeled probes were prepared from linearized templates for *fgf8, erm* and *pea3,* using an RNA labeling and detection kit (Roche). Stained embryos were dissected and mounted in glycerol.

### Cell Nuclei Visualization

After ISH staining, embryos were then incubated in 25 µM SYTOX nucleic acid stain (Invitrogen) overnight. After washing in 1×PBS embryos were mounted laterally for confocal analysis.

### Treatments/Chemical Treatments

Inhibition of Fgf receptor-mediated signaling was performed by incubating live embryos in 16 µM of SU5402 (Calbiochem) in 1% DMSO or with 1% DMSO only at 30% epiboly stage for a period of 3 hours and fixed at 75% epiboly stage for *in situ* hybridization experiments. Inhibition of molecular transport from the early to the late endosomes was performed by treatment of embryos with 100 nM bafilomycin A1 (Sigma-Aldrich) with 1% DMSO or 1% DMSO only for a period of 1 hour at 30% epiboly stage and subjected to live imaging or fixed at 75% epiboly stage for *in situ* hybridization experiments. Texas red Transferrin was purchased from Invitrogen.

### Cell Culture

Zebrafish PAC2 cells were used to study the intracellular localization of Fgf8. The following endosomal markers were used: RFP-Rab5a (human) from Ari Helenius, pCS2+mCherry-dmRab7 from Jim Smith, mRFP-Clathrin from Ernst Ungewickell, dsRed-LAMP1 from Erez Raz. Cells were transfected separately with Fgf8-GFP at a concentration of 1 µg and Hsc70 transfected along with mRFP-Clathrin, RFP-Rab5a, mCherry-Rab7 at a concentration of 600 ng in DMEM solution. 24 hours post transfection, Fgf8-GFP transfected cells were co-cultured with cells transfected with Hsc70 and the respective endosomal markers.

### Image Acquisition

Prior to imaging, embryos were mounted in 70% glycerol. Images were taken with the help of an Olympus SZX16 microscope equipped with a DP71 digital camera using the imaging software Cell A. For confocal analysis, embryos were embedded for live imaging in 1.5% low melting point agarose (Sigma-Aldrich) dissolved in 1x E3 solution at 50% epiboly stage. Live embryos as well as cells were images using 20x and 63x water immersion objective. Confocal image stacks were then obtained using the Leica TCS SP5 X confocal laser-scanning microscope. Images were further processed using Imaris 6 (Bitplane AG).

### Quantification

The areas for Fgf target gene expression *erm* and *pea3*, were then measured using Olympus Cell A software. The area of expression was calculated with respect to the area of the entire embryo. Confocal experiments were quantified using Imaris 6 (Bitplane AG).

## Results

### Hsc70 Regulates the Fgf8 Signaling Range

**Figure 1:**
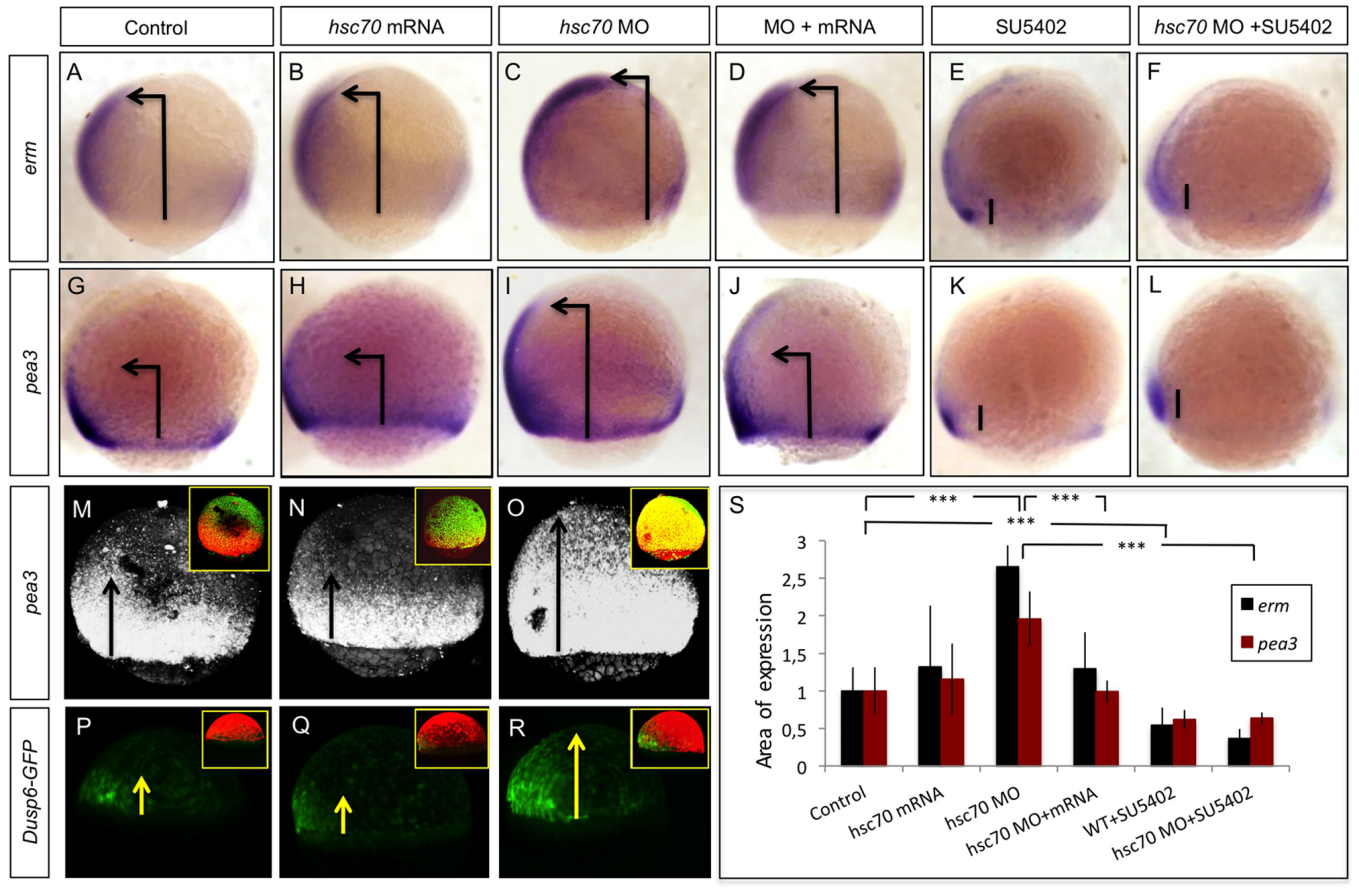
Hsc70 regulates the Fgf8 signaling range. (A–L) *In situ* hybridization for Fgf target genes *erm* and *pea3* at 75% epiboly stage. Embryos were mounted laterally with animal pole to the top and dorsal region towards the left. The area of the expression domains of Fgf target genes *erm* and *pea3* were investigated in embryos injected with indicated constructs and the border of the expression domains were marked by arrows. (E, F, K, L) show embryos co-treated with 16 µM of the Fgf signaling inhibitor SU5402. Fluorescent *ISH* for target gene expression *pea3* is shown in (M–O). As an *in-vivo* reporter for Fgf signaling, the expression of Dusp6-EGFP in live zebrafish embryos at 50% epiboly stage was investigated (P–R). The area of expression of target genes *erm* and *pea3* (A–L) was quantified in 10 embryos for each experiment (S).

To test if CME may interfere with Fgf8 signaling, we focused on Hsc70 mediated cycle of Clathrin. Therefore, we altered the level of Hsc70 function in zebrafish and analyzed the expression of bona-fide, high-sensitive target genes of Fgf8 signaling, *erm* and *pea3* by *in-situ* hybridization (ISH; [33]). We found that overexpression of *hsc70* mRNA did not change the expression area of the target genes *erm* and *pea3* compared with control embryos (Fig. 1A,B,G,H). To further analyze the function of Hsc70 we knocked-down its expression using two independent Morpholino (MO) antisense oligomers (Fig. S1). We found that down-regulation of Hsc70 expression led to an increase in the expression domain of both target genes (Fig. 1 C,I, arrows) suggesting an expanded Fgf8 signaling range. Co-expression of MO-insensitive ectopic *hsc70* mRNA rescued partially the expanded signaling range in morphant embryos (Fig. 1 D,J). To prove Fgf dependence of the expression of these two genes during alteration of Hsc70 function, we treated embryos with the Fgf signaling antagonist SU5402, which blocks specifically Fgfr phosphorylation without interfering with other growth factor receptors [34]. Treatment with the inhibitor reduced *erm* and *pea3* gene expression compared to control embryos and we observed a similar down-regulation in Hsc70 knocked-down embryos (Fig. 1 E,F,K,L). This suggests that Hsc70 function influences early Fgf receptor mediated signaling. Quantification of the area of expression of *erm* and *pea3* showed a significant increase in the *hsc70* knock-down embryos, whereas the area of expression was significantly reduced in SU5402 treated embryos (Fig. 1 S). In a second set of experiments we examined the range of signaling by a high-sensitive fluorescent ISH approach as well as in a stable transgenic zebrafish line carrying Dusp6-GFP as an *in-vivo* reporter for Fgf signaling [35]. Consistently, we found that both, *pea3* and Dusp6-GFP expression are expanded in *hsc70* morphant embryos (Fig. 1 M,O,P,R), while the expression areas of *pea3* and Dusp6-GFP in embryos overexpressing Hsc70 was unaltered (Fig. 1 N, Q). To exclude if knock-down or overexpression of Hsc70 had an effect on the expression of the ligand, we performed an ISH against Fgf8 (Figure S1 B–E). Indeed, we found that Fgf8 expression is not altered in the marginal zone in *hsc70* morphant embryos, however, in a few embryos the expression seems to be even reduced, suggesting that Hsc70 function is involved in regulating Fgf signaling mainly in the receiving tissue.

### Clathrin Mediated Endocytosis Regulates Fgf8 Internalization

**Figure 2:**
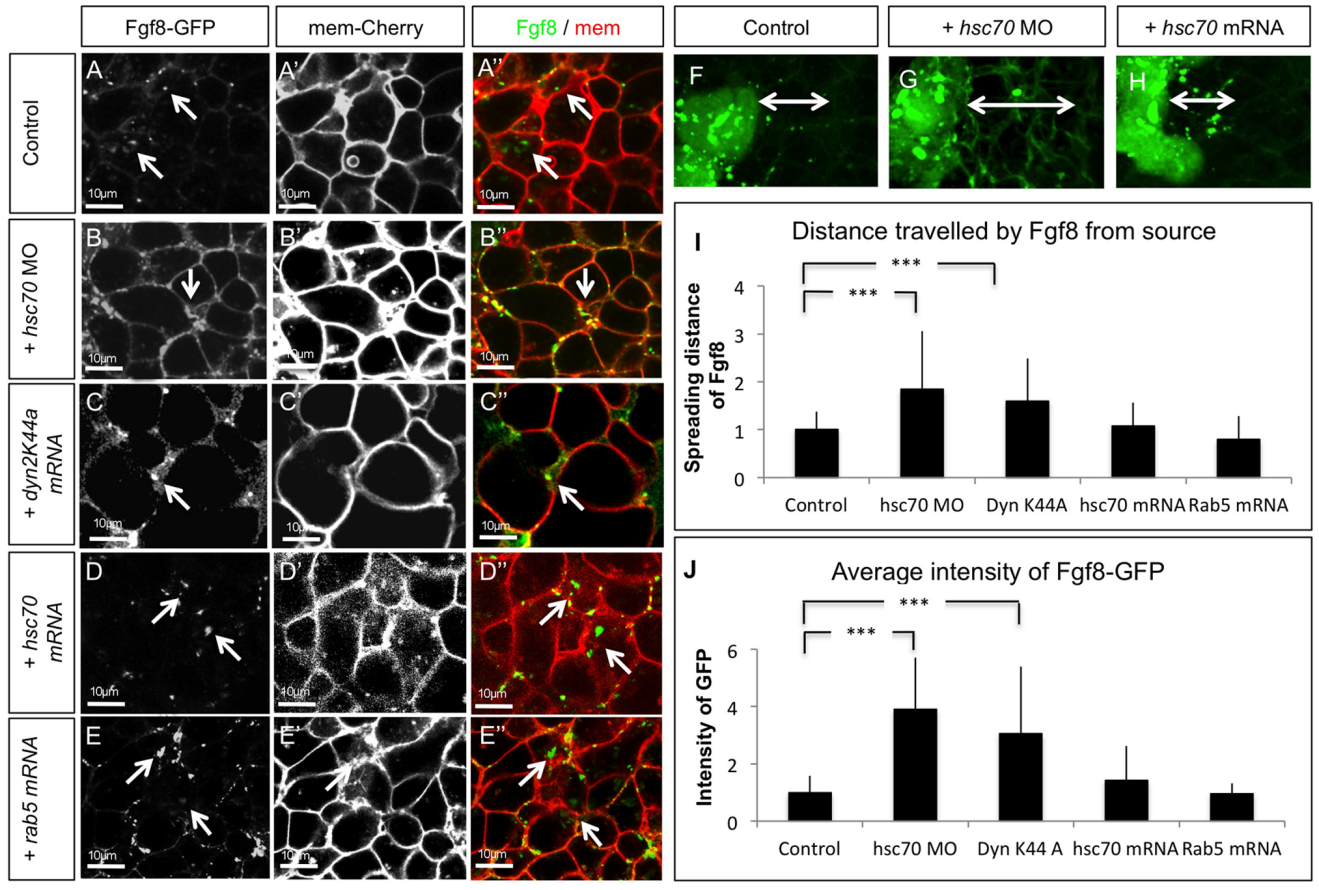
Fgf8 uptake is regulated by Hsc70. Confocal analysis of live embryos at 50% epiboly stage at the animal pole. Fgf8-GFP DNA was injected along with the red membrane marker mCherry at the one cell stage to determine subcellular localization of Fgf8 in embryos co-injected with indicated constructs (A–E”). The range of Fgf8 propagation in the receiving tissue was analyzed using confocal microscopy (F–H) and the distance spread by Fgf8 was quantified (I). Quantification of the average intensity of Fgf8-GFP is demonstrated in embryos (J). For quantification 7 different embryos were used for each experiment.

To address the question how Hsc70 function interferes with Fgf8 signaling, we visualized spreading and uptake of Fgf8 into cells in blastula-stage embryos by analyzing the distribution of a GFP-tagged form of Fgf8 [31] in the living embryo. Therefore we generated focal Fgf8 sources by microinjection of Fgf8-GFP DNA from which the signaling molecules travel to the neighboring cells and get internalized (Figure S2). In control embryos, we found Fgf8 localized at the membrane as well as in intracellular clusters of the receiving cells (Fig. 2 A–A”). Upon knock-down of Hsc70 function Fgf8-GFP accumulated in the extracellular space (ECS) and intracellular clustering was strongly reduced (Fig. 2 B–B”). In case of inhibition of endocytosis by blockage of Dynamin2 function, embryos showed a similar accumulation of Fgf8-GFP in the ECS (Fig. 2 C–C”). The extracellular accumulation of Fgf8 during loss of Hsc70 function suggests that blocking Hsc70 function leads to reduced rate of Fgf8 endocytosis. Indeed, it has been suggested that blockage of Hsc70 function leads to inhibition of uncoating of CCVs and thus reduces CME consequently [23], [24].

Consistently, overexpression of Hsc70 led to an enhanced intracellular clustering of Fgf8 and the signal in the ECS was decreased (Fig. 2D–D” ). Previously, it has been reported that activation of Rab5 controls the transport between the cell membrane and the early endosome [36], [37]. We found that increase of endocytosis resulting from overexpression of the GTPase Rab5 led to similar intracellular Fgf8 clustering (Fig. 2E–E” ) compared to Hsc70 overexpression.

Next, we measured the spreading range of Fgf8 from the producing cells in vivo (Fig. 2 F–H). We found Fgf8-GFP at a distance of about four cells from the source tissue (Fig. 2 F). After blockage of Hsc70 function or expression of the dominant negative Dyn2-K44A, Fgf8 spreading range increases to seven cells (Fig. 2 G). Hsc70 overexpressing embryos, as well as in embryos activated with Rab5 the spreading range is unaltered compared to the control embryos (Fig. 2 H). The quantification of these data is in support of our ISH based analysis of the endogenous Fgf8 signaling range (Fig. 2 I, Fig. 1).

Next, we quantified the fluorescence intensity of Fgf8-GFP to have an indirect measure of the influence of Hsc70 on Fgf8 stability in embryos (Fig. 2 J). We found that about 3 to 4 fold increased fluorescence intensity when Fgf8 internalization was blocked during knockdown of Hsc70 and Dyn K44A in comparison to control embryos. Overexpression of Hsc70 or Rab5 showed no significant difference in the fluorescence intensity when compared to control embryos. This data suggests that reduced internalization leads to an accumulation of Fgf8 due to reduced degradation. Based on these results we conclude that Hsc70 function is important for Fgf8 endocytosis followed by degradation.

### Hsc70 Expression Facilitates Cargo Uptake in Early Endosomes

**Figure 3:**
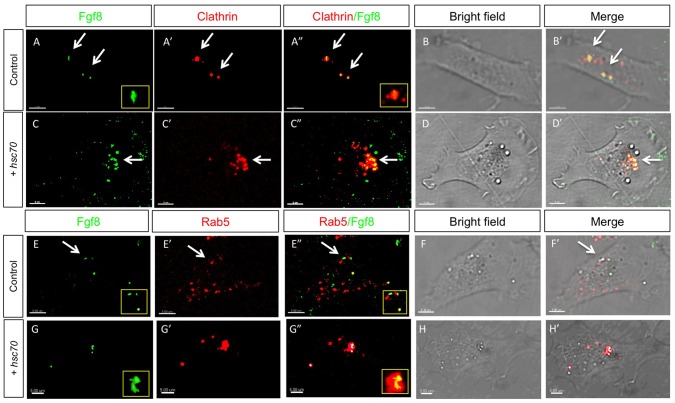
Analysis of Fgf8 internalization Clathrin coated vesicles and early endosomes in fish fibroblasts. Confocal analysis of Fgf8-GFP co-localization with indicated early endocytic markers in zebrafish PAC2 cells. Columns 1–3 show confocal images and columns 4–5 shows bright field images merged with the confocal image. Co-localization of Fgf8 with Clathrin (A–D’) and with Rab5 (E–H’) was investigated. Furthermore, insets show the increasing size of Rab5 positive early endosomes upon Hsc70 overexpression (E and G).

Next we wanted to address if Hsc70 promotes the uptake of cargo in specific endosomal compartments. To characterize the intracellular compartment affected by Hsc70 function, we established a cell culture assay in which we co-cultivated zebrafish PAC2 cells transfected with Fgf8-GFP (producing cells) and PAC2 cells transfected with endocytic markers (receiving cells; [38]). We transfected mRFP-Clathrin light chain A to mark Clathrin-coated vesicles and found a strong co-localization of clathrin with Fgf8 (Fig. 3 A–B’), suggesting that CME is the main uptake route for Fgf8 in zebrafish. After co-transfection of Clathrin and Hsc70 we found an increasing proportion of Fgf8 clusters that are negative for Clathrin (Fig. 3 C–D’, arrow), which were not observed in the control experiments. To mark early endosomes, we transfected Rab5-mCherry in the receiving cells (Fgf8 co-localized to Rab5 positive vesicles only in a few cases (Fig. 3 E–F’), suggesting that Fgf8 localization to early endosome is a transient stage. However, activation of Hsc70 in the same cells led to the formation of Fgf8 clusters, which strongly co-localized with Rab5 positive early endosomes (Fig. 3 G–H’), suggesting that activation of Hsc70 lead to an efficient recruitment of Fgf8 into early endosomes.

**Figure 4:**
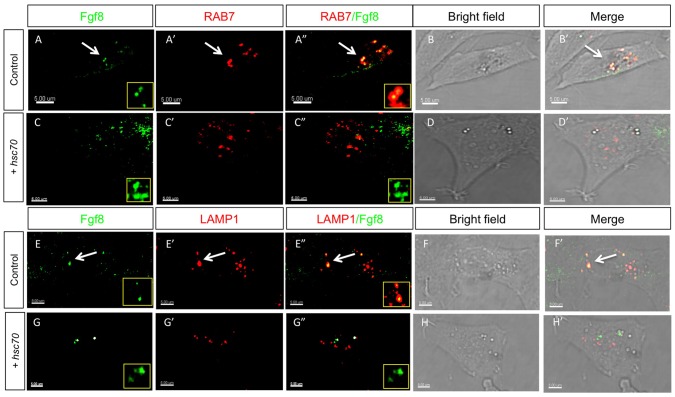
Analysis of Fgf8 internalization in late endosomes and lysosomes in fish fibroblasts. Confocal analysis of Fgf8-GFP localization in zebrafish PAC2 cells. Columns 1–3 show confocal images and columns 4–5 shows bright field images merged with the confocal image. Co-localization with late endosomal markers was investigated: with Rab7 (A–D’) and the lysosomal Lamp1 (E–H’).

Next, we marked late endosomes with Rab7-mCherry and indeed, we found co-localization of Fgf8 with Rab7 (Fig. 4 A–B’). However, Hsc70-induced Fgf8 clusters did not co-localize with Rab7 positive vesicles (Fig. 4 C–D’). To visualize lysosomes we used Lamp1 and we found co-localization with Fgf8 and Lamp1 (Fig. 4 E–F’). Similar to Rab7, co-localization of Lamp1 and Fgf8 is strongly reduced upon Hsc70 activation (Fig. 4 G–H’).

**Figure 5:**
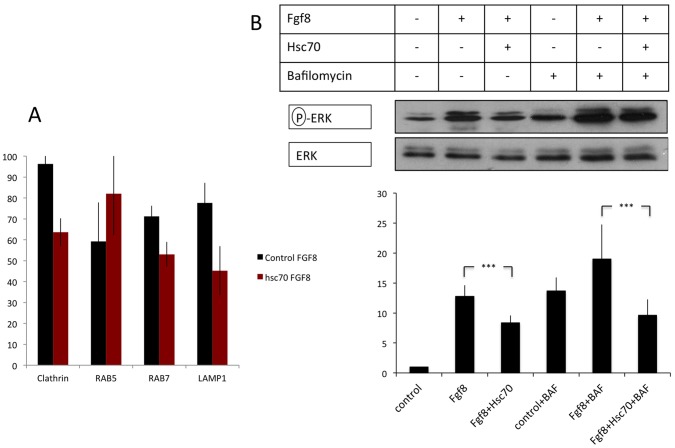
Quantification of localization and signaling during endocytosis. Co-localization of Fgf8 is found with Clathrin, Rab7 and Lamp1, whereas co-localization of Rab5 is reduced (A). However, after stimulation of Hsc70, co-localization of Fgf8 and Rab5 is strongly increased. Three independent experiments were quantified. Cultivation of HEK293 cells after transfection with Fgf8, and Hsc70 with and without treatment of Bafilomycin A1 (B). Three independent experiments were analyzed and the ratio between activated Erk1/2 and Erk total was calculated. Cells transfected with Fgf8 showed a significant 12-fold increase in Erk1/2 phosphorylation compared to the un-transfected control. Co-transfection of Hsc70 and Fgf8 led to 8-fold increase of phosphorylation of Erk. Treatment with Bafilomycin A1 leads to a 14-fold increase of double phosphorylated Erk1/2 level. Activation by co-transfection of Fgf8 leads to a significant 19-fold increase, and co-transfection of Hsc70 and Fgf8 decreased this activation to a 10-fold activation of double phosphorylated Erk.

Next we quantified the performed our co-localization studies of Fgf8-GFP with endocytic vesicle markers. We found that Fgf8 is mainly localized to Clathrin-coated vesicles (96%), late endosomes (72%) and lysosomes (78%; Fig. 5 A). Activation of Hsc70 forces Fgf8 significantly to accumulate in Rab5 positive early endosomes (81%), whereas co-localization with all other examined endocytic markers is reduced. In addition, we analyzed the co-localization of Fgf8 clusters with a diameter bigger 0.8 µm in Hsc70 transfected PAC2 cells (Figure S4). We found that the majority of Fgf8 clusters (88%) co-localize to Rab5 positive vesicles, whereas only a minority of the clusters co-localize to Clathrin (42%), Rab7 (48%), Lamp1 (31%). This suggests that Hsc70 activation leads to an efficient sequestering of Fgf8 to Rab5 positive early endosomes.

### Transport of Fgf8 from Early to Late Endosomes Controls Signaling

We next analyze the consequence of Fgf8 endocytosis for signaling. We used a cell cultivation assay in which we analyzed effective Fgf8 signaling by quantification of Erk1 and Erk2 phosphorylation levels. Transfection of Fgf8 led to a 12-fold stronger level of Erk1/2 phosphorylation compared to the unstimulated control cell culture (Fig. 5B). Simultaneous activation of Hsc70 and Fgf8 reduced the activation to 8 fold. Next, we blocked cargo transport from early endosomes to late endosomes by treatment with Bafilomycin A1 [39]. We observed that treatment with Bafilomycin A1 led to a 14-fold stronger Erk1/2 phosphorylation compared to the control experiment. Co-transfection of Fgf8 increased the magnitude of this effect to a 19-fold activation. Similar to the experiments without Bafilomycin A1 treatment, co-transfection of Hsc70 and Fgf8 showed a decrease in Erk1/2 phosphorylation to a 10-fold activation. These experiments suggest that plasma membrane and early endosome are important signaling compartments for Fgf8. Reduction of signaling by co-transfection with Hsc70 suggests that Fgf8 signaling is exerted predominately at the plasma membrane and not in early endosomes. An increase in signaling due to Bafilomycin treatment suggests that the transport of the activated (Fgf8-Fgfr) complex from early endosomes to late endosomes is the key step to limit Fgf8 signaling strength. Similarly after Bafilomycin treatment, effective translocation of Fgf8 from the plasma membrane into early endosomes by co-transfection of Hsc70 leads to a down-regulation of signaling supporting our data that Fgf8 signal transduction is mainly exerted at the plasma membrane.

### Fgf8 Spreading Range is Independent of Transport from Early to Late Endosomes

**Figure 6:**
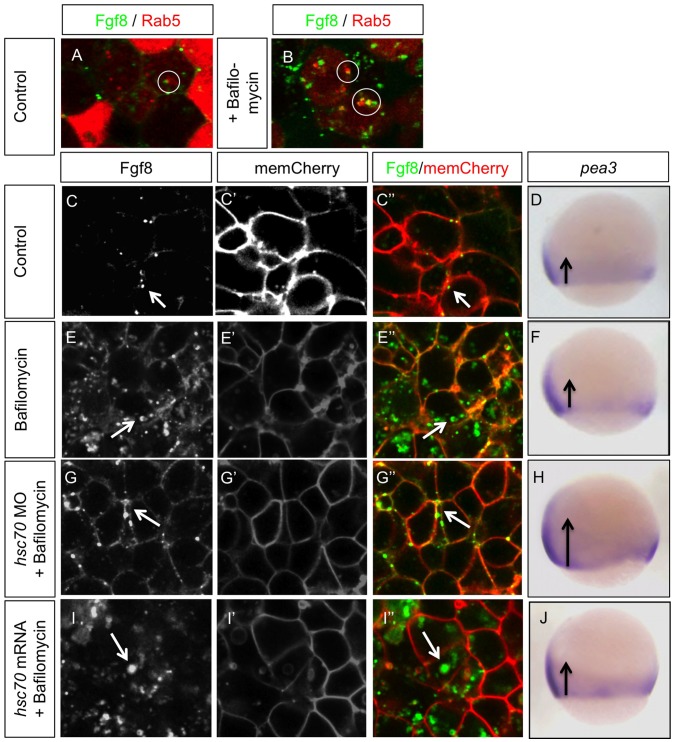
Inhibition of Fgf8 transport from early to late endosome does not alter signaling range. Analysis of localization of Fgf8-GFP during inhibition of endocytic transport from early to late endosomes by treatment with Bafilomycin A1. Embryos were injected with Fgf8-GFP along with the early endosomal marker Rab5 or membrane marker mCherry at one cell stage, treated with 100 nM Bafilomycin A1 at 30% epiboly stage for a period of 1 hr and subjected to live imaging using confocal microscopy at 50% epiboly stage. At 75% epiboly stage embryos were fixed and stained for *erm* expression by ISH. Circle highlight co-localization of Rab5 and Fgf8, white arrows point to typical Fgf8 localizations, and balck arrows visualize the extend of the *pea3* expression domain.

Based on the data from the cell culture experiments we tested our hypothesis if the transport of Fgf8 along the endocytic route influences the signaling range. First we analyzed the endocytic route of Fgf8 in zebrafish embryos. We found that Fgf8 was taken up into Rab5 positive early endosomes, however, co-localization of Rab5 and Fgf8 was a rare event (Fig. 6A) supporting our data in cell culture (Fig. 2). We hypothesized that endocytosis of Fgf8 leads to a fast degradation of the ligand. To validate this hypothesis, we treated embryos with Bafilomycin A1 to block maturation of early to late endosomes and analyzed the localization of Fgf8. Indeed, we found that treatment with Bafilomycin A1 increased the localization of Fgf8 in Rab5 early endosomes (Fig. 6B). Next, we addressed if endocytic routing has an influence on the signaling range determining the morphogenetic field of Fgf8. We found that Bafilomycin A1 increased the amount of Fgf8 in early endosomes compared to the control embryos (Fig. 6 C–C”, E–E”). The signaling range of Fgf8 in Bafilomycin A1 treated embryos was unaltered when compared with the control embryos (Fig. 6 D, F), suggesting that the blockage of transport from early to late endosome does not affect CME and therefore the signaling range. Simultaneous knock-down of Hsc70 and Bafilomycin A1 treatment led to a localization of Fgf8 in the extracellular space (Fig. 6 G–G”) and increased signaling range (Fig. 6 H), comparable to Hsc70 morphant embryos (Fig. 1 & 2). This suggests that blockage of Hsc70 leads to attenuation of Fgf8 internalization and, therefore, Fgf8 degradation is reduced. Expression of Hsc70 mRNA and Bafilomycin A1 treatment led to a decrease in extracellular Fgf8, supporting the idea that Hsc70 overexpression increases Fgf8 uptake when compared to Bafilomycin A1-only treated embryos. Consistently, we found strong intracellular accumulation of Fgf8 during Hsc70 overexpression (Fig. 6 I–I”), suggesting that Fgf8 is taken up more quickly in early endosomes and the further degradation route is pharmacologically attenuated. In these embryos, we found that the signaling range is unaltered (Fig. 6 J). Therefore, we conclude that blockage of Fgf8 transport from early endosomes to late endosomes does not change the uptake nor the signaling range of Fgf8, however, it influences Fgf8 signaling strength as supported by the previous experiment (Fig. 5).

## Discussion

### Fgfs and Clathrin-mediated Endocytosis (CME)

**Figure 7:**
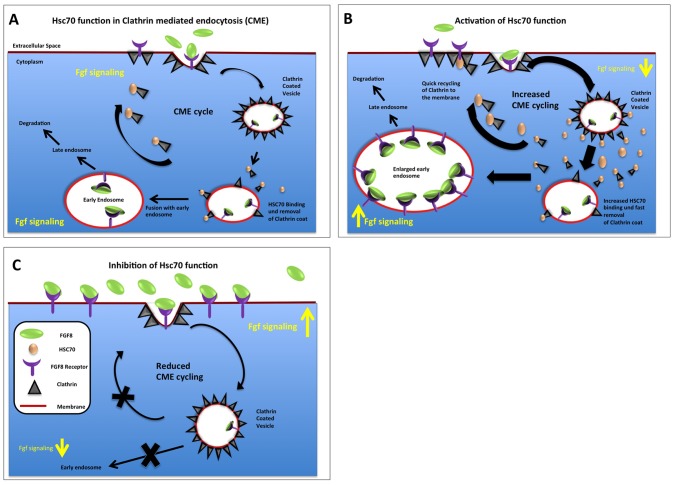
Graphical summary of Hsc70 function regarding Fgf8 endocytosis in zebrafish. Detailed explanation in text.

Although Fgf-Fgfr interaction has been extensively studied, the internalization [40] pathways and their effects on signaling remain unclear. In this study we demonstrated that endocytosis of Fgf8 depends on CME in zebrafish (Fig. 7A). Indeed, there are numerous examples showing that Fgfr internalization employs CME. Furthermore, Fgfr3 and Fgfr2b (formerly known as keratinocyte growth factor receptor; KGFR) localize to clathrin-coated pits and depletion of clathrin inhibits Fgfr2 endocytosis [40]. Furthermore, cells depleted of clathrin heavy chain show stabilization of active Fgfr1 at the plasma membrane [41]. Recent data from zebrafish suggest that in addition to CME, Fgf8 may be alternatively internalized via Caveolae [17].

We hypothesized that the rate of CME alters the intracellular uptake of Fgf8 and therefore determines the extracellular availability of Fgf8 and, hence the signaling range (Fig. 1 & 2). Previously, it had been suggested that endocytosis is a crucial mechanism in defining signaling range. Blockage of Fgf8 endocytosis by inactivation of Rab5 with RNTre [16] or blockage of ubiquitination of the receptors [17] prolongs the retention time of the active Fgf8-Fgfr complex at the membrane. As a consequence continuous Fgf8 removal from the extracellular space is attenuated and extracellular Fgf8 concentration increases. This subsequently leads to a pronounced spreading of the signaling molecule from its source thereby increasing the signaling range. In support of this result, we found that knock-down of Hsc70 prevents endocytosis of Fgf8 (Fig. 2, Fig. 7C) leading to an increased range of activation (Fig. 1). It has been shown previously that in order for a diffusive morphogen gradient to attain a steady state, there needs to be a ligand “sink” [42], [43]. In this study we suggest that uptake of Fgf8 by CME is required for its removal from the extracellular space, preventing the increase in the signaling range, thereby controlling the formation of a morphogen gradient as postulated in the ‘restrictive clearance model’ [4], [16], [31].

### Blockage of CME Leads to an Increase in Fgf Signaling Range

In parallel, our experiments suggest that Fgf8 signaling can occur at the cell membrane and to a lesser extend in Rab5 positive early endosomes, which contrasts with other growth factor signaling pathways. As a result of impaired CME by expression of Dynamin2 K44A, activated Egfr shows a prolonged residence at the plasma membrane, which leads to a reduced activity of downstream signaling components such as MAPKs Erk1/2 or the p85 subunit of phosphatidylinositol 3 kinase, PI3 kinase [44]. However, for Fgf signaling it has been shown that cells depleted of clathrin heavy chain show stabilization of the active Fgfr1 at the plasma membrane which increases signaling [41]. Furthermore, increasing the half-life of the active Fgfr at the membrane by blockage of ubiquitination leads to a similar increase in signaling [17]. A second possibility is that CME might also play a role in endosomal sorting and recycling [45], [46]. Therefore we hypothesize that signaling from the receptors is prolonged upon blockage of CME due to both inefficient internalization and inefficient sorting to lysosomes. However, we found that increased CME changed the localization but not the concentration of Fgf8 (Figure 2 & 8B). This suggests that the uptake of Fgf8 is an independent process and does not interfere with the subsequent transport of Fgf8 from early to late endosomes. Interestingly, cells activated for Hsc70 showed no obvious decrease in the signaling range. This could be due to enhanced cell migration (epiboly) during the developmental stage investigated and the expansion reflects the migratory activity of the *erm* and *pea3* positive cells rather than Fgf8 spreading. An increased rate of endocytosis uncoupled from cell migration leads to a decrease of the spreading range [16]. Interestingly, cells with knocked down Clathrin showed a delayed Fgf signaling [41]. One explanation here could be that extracellular degradation of Fgf8 may act as a further mechanism to control signaling. In other morphogen signaling systems extracellular ligand degradation is a common theme: Wnt3a is inactivated by the protease Tiki [47] and Chordin by the metalloprotease Tolloid [48]. Recent reports have highlighted the importance of extracellular inhibitors also for Fgf signaling, too [49], [50]. A second hypothesis is that translocation of the activated Fgfr from the plasma membrane to the early endosomes enables other adaptor proteins to bind to the receptor, which transduce signaling via a different mechanism. Indeed, the Ap2 endocytic complex which links the Clathrin coat to internalized cargo can only be found at the plasma membrane and is shed off prior to vesicle fusion with the early endosome [51], which opens up the possibility of interactions with other adaptor proteins such as Eps8 and Src [52].

### Translocation of Fgf8 from the EE to the LE Terminates Signaling

After CME, Fgf8 localizes to early endosomes and late endosomes. Blockage of Fgf8 transport to late endosomes increases signaling. This suggests that - in addition to signaling at the membrane - signaling occurs also from early endosomes. Indeed the hypothesis that Fgf signaling can occur through the entire endocytic route has been postulated already in the 80s [53], [54]. The hypothesis that signaling from receptor molecules occurs not only at the plasma membrane but also from internalized ligand-receptor complexes has been recently supported: KGF and KGFR were found to remain associated in active complexes through the endocytic pathway [40], [55] and activated Fgfr4 was found in the compartment of recycling endosomes [27]. However, Fgfr internalization subsequently leads to degradation of the receptor within minutes [40], [56], [57], [58]. Indeed, we find that co-localization of Fgf8 to Rab5 early endosomes is a fast step whereas Fgf8 localization in late endosomes and lysosomes is clearly detectable (Fig. 3 & 5). It has been shown that binding of Fgf induces ubiquitination of Fgfr1 and Fgfr3, and this contributes to receptor down regulation [17], [59], [60], [61]. Fgfr was found to recruit the ubiquitin ligase Cbl by an indirect mechanism involving the docking protein Frs2a and Grb2 [60]. Our co-localization studies with intracellular markers showed that Hsc70 over-expression leads to an efficient translocation of Fgf8 from the cell membrane into early endosomes (Fig. 3 & 4). Furthermore, we found that Hsc70 mediated endocytosis reduces signaling (Fig. 5) suggesting that Fgf8 signaling occurs predominately at the plasma membrane. To analyze the contribution of the early endosome to signaling and the late endosome to degradation we blocked endosome maturation pharmacologically. Bafilomycin A1 blocks acidification of early endosomes by inhibition of V-ATPase activity [62], and therefore inhibits Arf6/ARNO mediated cargo transport from early endosomes to late endosomes [39]. We found that treatment with Bafilomycin A1 blocked Fgf8 degradation (Fig. 6), which leads to an up-regulation of Fgf8 signaling consequently (Fig. 5), suggesting that Fgf8 translocation from the early endosome to the late endosome terminates signaling. In summary, we show that accumulation at the plasma membrane and inhibition of degradation of Fgf8 in late endosome by Bafilomycin results in an increase in the signaling strength/activity of Fgf8 determined by the increase in the expression of phosphorylated Erk (Fig. 5). We hypothesized that the average residence time of activated Fgf8-Fgfr complex mainly at the plasma membrane and to a lesser extend in the early endosome defines the level of activation of the Fgf pathway. It has been suggested that receptors in endosomes can recruit and activate other downstream signaling molecules when compared to receptors at the cell surface [44]. Indeed, stimulated uptake of Fgf8 and simultaneous blockage of the transition to late endosomes decreased signaling (Figure 5 & 7), suggesting that the main signaling compartment is the plasma membrane. Complete phosphorylation of Fgfr and of downstream signaling molecules were reached at a later time point in clathrin-depleted cells than in non-depleted cells [41]. This indicates that endocytic trafficking may regulate the timing of signaling.

### Conclusion

Here we conclude that the dynamics of Clathrin-mediated endocytosis of Fgf8-Fgfr complexes regulates the signaling range of Fgf8, whereas the retention time of the activated signaling complex at the plasma membrane and in the early endosome defines signaling strength.

## Supporting Information

Figure S1 A. Immunoblot analysis of the expression of Hsc70 protein during knockdown and over expression of Hsc70. Embryos were injected with Hsc70 morpholinos as well as Hsc70 mRNA and samples at 28hpf were subjected to immunoblot analysis. Embryos injected with Hsc70 MOs showed a reduction in the expression of Hsc70 when compared to the expression in wild type samples. Embryos overexpressing Hsc70 showed an increase in the expression of Hsc70 when compared to the expression in the wild type. ISH for Fgf8 expression. (B) Shows the expression of Fgf8 at 75% epiboly stage. Fgf8 expression remains unaltered during overexpression (C), knock-down of Hsc70 (D), and injection of Hsc70 MO and mRNA (E).
(TIF)

Figure S2 **Endocytosis of Transferrin.** Transferrin is taken up into the endosomes in fish fibroblasts (A–A”). Activation of Hsc70 leads to the formation of clusters of Transferrion, most likely in early endosomes (B–B”).
(TIF)

Figure S3 **Injection procedure to measure the distance of Fgf8 distribution.** Figure shows the schematic diagram of the experiment performed to analyze the spreading of Fgf8 from its source. (A) Fgf8 GFP DNA was injected into live zebrafish embryos at the one cell stage. (B) Zebrafish embryo at 50% epiboly stage show a mosaic expression of Fgf8. (C) Confocal microscopy analysis of live embryos show Fgf8 expressed in specific cells creating local sources from which the distance travelled by Fgf8 from the source to receiving cells is measured (e.g. a,b).
(TIF)

Figure S4 **Quantification of co-localization studies of Fgf8 with endosomal markers.** Transfection of Hsc70 led to the formation of Fgf8 clusters with a diameters over 0.8 µm. These clusters co-localize similarly strongly in Rab5 positive early endosomes. Three independent experiments were quantified.
(TIF)
