# Supplementary figures and images for: Endocytosis of Fgf8 Is a Double-Stage Process and Regulates Spreading and Signaling
Source: PLoS One. 2014 Jan 20;9(1):e86373. doi: 10.1371/journal.pone.0086373 (PMC3896487; doi:10.1371/journal.pone.0086373)

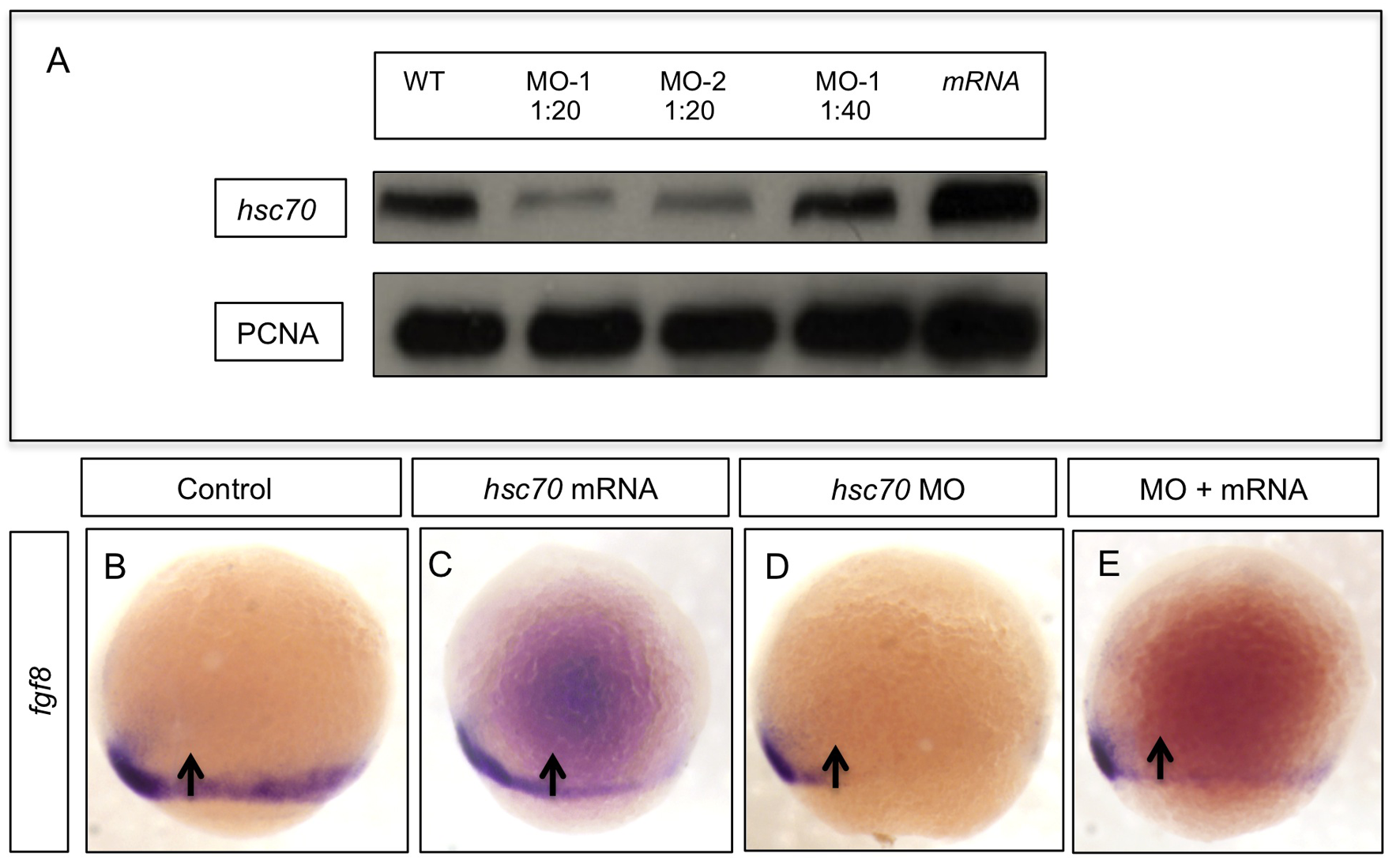

Supplement: Figure S1 — A. Immunoblot analysis of the expression of Hsc70 protein during knockdown and over expression of Hsc70. Embryos were injected with Hsc70 morpholinos as well as Hsc70 mRNA and samples at 28hpf were subjected to immunoblot analysis. Embryos injected with Hsc70 MOs showed a reduction in the expression of Hsc70 when compared to the expression in wild type samples. Embryos overexpressing Hsc70 showed an increase in the expression of Hsc70 when compared to the expression in the wild type. ISH for Fgf8 expression. (B) Shows the expression of Fgf8 at 75% epiboly stage. Fgf8 expression remains unaltered during overexpression (C), knock-down of Hsc70 (D), and injection of Hsc70 MO and mRNA (E). (TIF) [file pone.0086373.s001.tif]

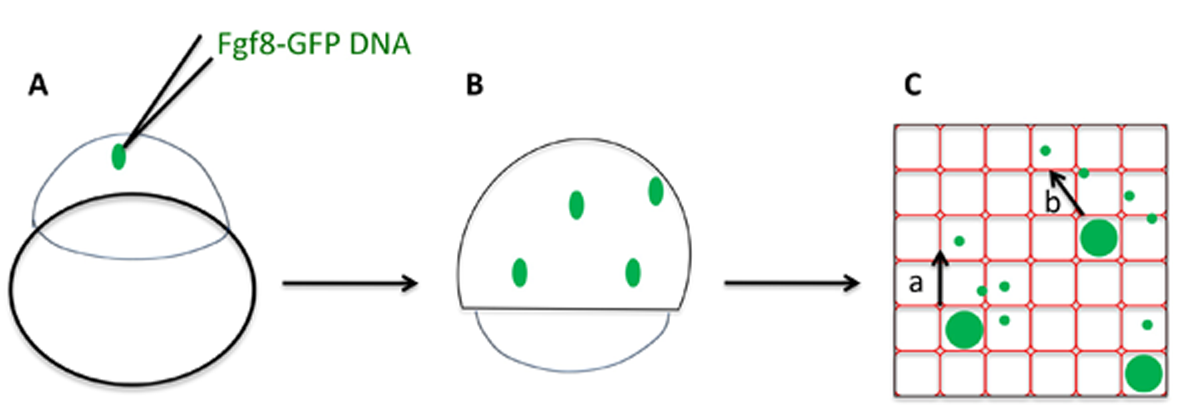

Supplement: Figure S2 — Endocytosis of Transferrin. Transferrin is taken up into the endosomes in fish fibroblasts (A–A”). Activation of Hsc70 leads to the formation of clusters of Transferrion, most likely in early endosomes (B–B”). (TIF) [file pone.0086373.s002.tif]

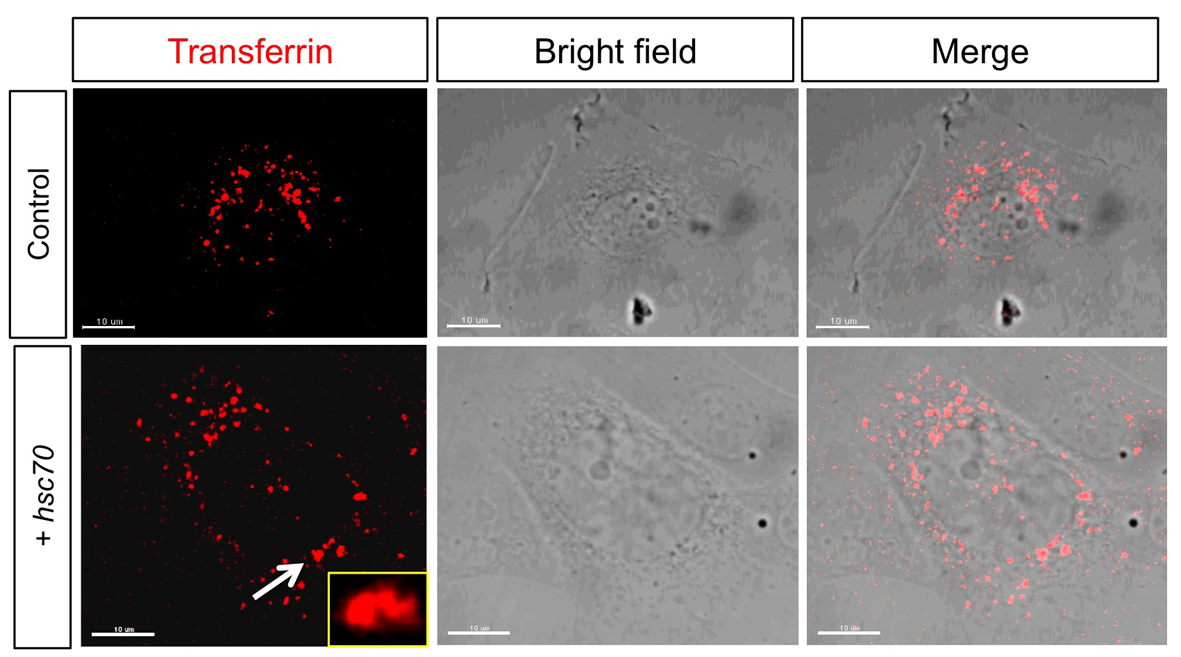

Supplement: Figure S3 — Injection procedure to measure the distance of Fgf8 distribution. Figure shows the schematic diagram of the experiment performed to analyze the spreading of Fgf8 from its source. (A) Fgf8 GFP DNA was injected into live zebrafish embryos at the one cell stage. (B) Zebrafish embryo at 50% epiboly stage show a mosaic expression of Fgf8. (C) Confocal microscopy analysis of live embryos show Fgf8 expressed in specific cells creating local sources from which the distance travelled by Fgf8 from the source to receiving cells is measured (e.g. a,b). (TIF) [file pone.0086373.s003.tif]

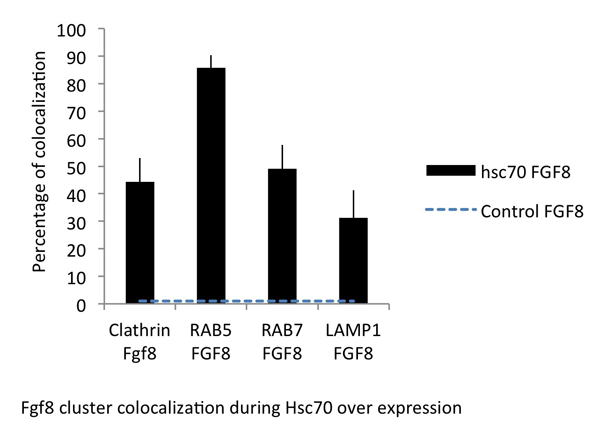

Supplement: Figure S4 — Quantification of co-localization studies of Fgf8 with endosomal markers. Transfection of Hsc70 led to the formation of Fgf8 clusters with a diameters over 0.8 µm. These clusters co-localize similarly strongly in Rab5 positive early endosomes. Three independent experiments were quantified. (TIF) [file pone.0086373.s004.tif]
